# Factors That Govern the Induction of Long-Lived Antibody Responses

**DOI:** 10.3390/v12010074

**Published:** 2020-01-07

**Authors:** Bryce Chackerian, David S. Peabody

**Affiliations:** Department of Molecular Genetics and Microbiology, University of New Mexico School of Medicine, Albuquerque, NM 87106, USA; dpeabody@salud.unm.edu

**Keywords:** long-lived plasma cells, antibodies, multivalency, virus-like particles

## Abstract

The induction of long-lasting, high-titer antibody responses is critical to the efficacy of many vaccines. The ability to produce durable antibody responses is governed by the generation of the terminally differentiated antibody-secreting B cells known as long-lived plasma cells (LLPCs). Once induced, LLPCs likely persist for decades, providing long-term protection against infection. The factors that control the generation of this important class of B cells are beginning to emerge. In particular, antigens with highly dense, multivalent structures are especially effective. Here we describe some pathogens for which the induction of long-lived antibodies is particularly important, and discuss the basis for the extraordinary ability of multivalent antigens to drive differentiation of naïve B cells to LLPCs.

## 1. Introduction—B-Cell Responses to Vaccination

Most of the effective vaccines in use today work by inducing humoral immune responses. Activation of naïve B cells by vaccine antigens leads to a series of events which ultimately result in the production of memory B cells and differentiation into two classes of antibody secreting cells, short-lived plasmablasts and long-lived plasma cells (LLPCs). The induction of immunological memory is classically invoked to explain the effectiveness of many vaccines. Memory B cells express the membrane-bound B-cell receptor (BCR) and are primed to respond to antigen exposure. They emerge early from germinal centers and often their immunoglobulin molecules have already undergone isotype switching and some degree of affinity maturation, meaning that they can potentially produce more effective antibodies upon re-stimulation. Memory of vaccination can increase the magnitude of the antibody response and drive the more rapid production of class-switched, high affinity antibodies. Thus, for many pathogens, the existence of memory cells provides a kinetic upperhand that tips the balance from the pathogen to the immune system.

Nevertheless, there are a number of important pathogens for which vaccine-based induction of memory cells alone is insufficient to provide protective immunity. In these cases, protection depends on a pre-existing level of antibodies high enough to entirely prevent the initial infection event, because once one of these agents initiates an infection, it establishes a beachhead which is then partially or completely resistant to antibody effector activity. Consider three common human pathogens, human immunodeficiency virus (HIV), human papillomavirus (HPV), and *Plasmodium*, as examples of diseases in which an effective vaccine is dependent on the ability to induce high-titer and long-lasting antibodies: (i) Once transmitted, HIV quickly traffics to regional lymph nodes and other immune tissues where it can infect T cells and macrophages [1]. The HIV provirus integrates into the genomes of these infected cells, where it establishes a persistent virus reservoir. While anti-retroviral drugs can successfully control active HIV infection, this latent reservoir is refractory to both antiretroviral drugs and anti-HIV antibodies, allowing the virus to reemerge if drugs are discontinued. Thus, antibodies can potentially control infection, but cannot eliminate HIV, so prevention of initial infection is critical. (ii) Similarly, after HPV infects its target cells, basal epithelial cells, the virus remains intracellular and not accessible to antibodies. The HPV lifecycle is intimately tied to the differentiation of basal cells into terminally differentiated epithelial cells [2]. Only when these cells differentiate are HPV virions produced and released. However, HPV-associated cancers do not result from productive infection. HPV can also establish latency in infected cells, and, in some cases, integration or episomal maintenance of the HPV genome can lead to dysregulated expression of the viral oncogenes, E6 and E7 [3]. These HPV-infected basal epithelial cells are the origin of neoplastic lesions that can ultimately progress to invasive tumors. Thus, once HPV infection occurs, antibodies have no value in protecting against cancer [4]. Accordingly, vaccines that elicit HPV-neutralizing antibodies can protect against initial infection, but fail to induce the regression of previously established lesions [5]. (iii) Liver infection by the *Plasmodium* parasite, which causes malaria, occurs within a few hours of feeding by an infected *Anopheles* mosquito. Injected sporozoites rapidly migrate through the bloodstream to the liver where they infect hepatocytes [6]. Once inside hepatocytes, *Plasmodium* is resistant to the effects of anti-sporozoite antibodies, meaning that antibodies only have a brief window to exert their effects. Thus, for these (and many other) pathogens, vaccines can be effective only if they elicit neutralizing antibodies maintained at high levels over time. For these pathogens, vaccine-mediated induction of LLPCs is required.

## 2. The Molecular Events That Mediate B-Cell Activation and the Role of Multivalency 

The activation of B cells and the subsequent downstream events that result in antibody production are consequences of the initial interaction between an antigen and the BCR. The signaling events initiated by this interaction, which are the subject of many excellent reviews [7,8,9], stimulate B cell proliferation and upregulate MHC Class II and the costimulatory molecules that permit subsequent interactions with T helper cells. B-cell activation is a quantitative phenomenon, in which the degree of activation is dependent on both the affinity of the BCR for its cognate antigen [10,11] and the valency of the antigen. The critical role of antigen valency in B-cell responses was first recognized by Renee and Howard Dintzis at the Johns Hopkins School of Medicine [12,13,14], who assessed the immunogenicity of a T-cell independent antigen consisting of a polymer (polyacrylamide) displaying a model hapten (dinitrophenol; DNP). The modular nature of this system allowed the dissection of antibody responses as a function of the valency and density of DNP display. They concluded that “…the fundamental molecular event in the induction of the primary immune response is the linking together by a single antigen molecule of a critical number of separate hapten receptors into a molecularly connected entity”, which they termed an immunon [12]. Numerous subsequent studies established this relationship between antigen valency and B-cell responsiveness, particularly in the context of T-cell-independent antibody responses [15,16].

The series of events that begins with the activation of naïve B cells by a T-cell-dependent antigen and ultimately result in differentiation to LLPCs are more complicated than what occurs with a T-cell independent antigen, yet antigen valency and density also play an important role in this process. Multivalent interactions promote BCR clustering and the formation of lipid rafts [17,18,19,20]. These, in turn, promote signaling to the B cell and receptor-mediated internalization of the antigen complex [21], steps critical for B cells to present antigen on MHC Class II and receive help from CD4 T cells. Accordingly, multivalency enhances BCR clustering [22], BCR/antigen internalization and antigen presentation [23], as well as the upregulation of costimulatory molecules that are important for subsequent interactions with T helper cells [24]. Thus, these multivalent interactions have a profound effect on early steps in B-cell activation and ultimately influence antibody production and other downstream events.

While the influence of multivalency on the early steps in B-cell activation have been extensively studied, less is known about how these events influence the establishment of germinal centers (GCs) and production of LLPCs. GCs are discrete anatomical sites within B-cell follicles in which B cells proliferate and undergo somatic hypermutation and affinity maturation. It is here that they differentiate to memory cells and LLPCs. In GCs, B cells compete for binding with antigens displayed on follicular dendritic cells, and then present antigens to follicular T helper cells, which in turn provide survival signals to the B cell. Although this process does not require a multivalent antigen, enhanced B-cell crosslinking in GCs leads to increased GC B-cell proliferation and promotes differentiation to plasma cells, particularly in scenarios in which T help is limiting [25]. Thus, it is likely that multivalency can exert its stimulatory effects at multiple steps during the B-cell activation and differentiation process. 

## 3. Immune Responses Elicited by Multivalent Vaccines

The basic immunological studies described above were initiated, in part, to explain the potent immunogenicity of multivalent antigens in a vaccine setting. Many different multivalent display strategies have been employed. They include synthetic nanoparticles [26,27], liposomes [28], micelles [29], and polymers [30]. But one of the most common is to display antigens on platforms based on virus-like particles (VLPs). Many viral proteins have an intrinsic ability, when overexpressed, to self-assemble into VLPs. VLPs lack viral genomes, meaning that they are absolutely non-infectious. VLPs can be derived from diverse viruses, including viruses that infect humans, animals, insects, plants, and bacteria (for recent reviews on this subject, see [31,32] and elsewhere in this Special Issue of *Viruses*). VLPs intrinsically display their own antigens in highly dense, repetitive arrays, and they can also be used as platforms for the high valency display of heterologous antigens. Their multivalency is not the only feature contributing to VLP immunogenicity. Most VLPs have a diameter (between 10 and 100–200 nm) that is optimal for uptake into the lymphatic system. Entry into the lymphatics promotes trafficking to regional lymph nodes, deposition in the subcapsular spaces of the lymph nodes that are key sites for B-cell activation, and interactions with antigen-presenting cells, such as dendritic cells (DCs) [33]. Some VLPs (and some engineered nanoparticles as well) also interact with soluble components of the innate immune system to facilitate this trafficking [34,35]. Moreover, VLPs fall into the size range (<500 nm in diameter) of particulate antigens most readily taken up by DCs [36]. Many VLPs, natural [37] or engineered [38], package adjuvants that can also enhance immunogenicity. Lastly, because they are protein-based, VLPs are naturally a rich source of T helper epitopes (or can be engineered to be so [39]), which are critical for the induction of T-cell-dependent antibody responses. Taken together, these immunological features underlie the use of VLPs as effective stand-alone vaccines (against the virus that they were derived from) and as the basis of vaccine platform technologies to display heterologous antigen targets. 

**Figure 1:**
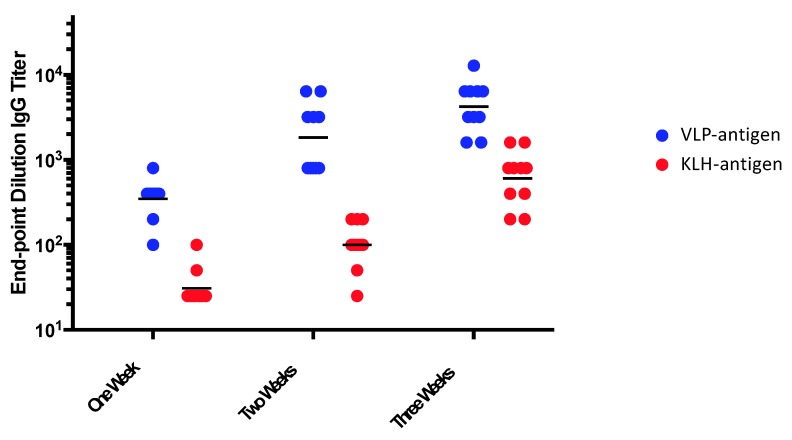
Mice were immunized with a single dose of an EGFRvIII peptide conjugated to Qß VLPs or the same peptide conjugated to keyhole limpet hemocyanin (KLH). Mice were immunized with 5 µg of VLP-EGFRvIII (displaying ~270 EGFvIII peptides per 2520kD VLP) or 25 µg of KLH-EGFRvIII (displaying ~50 EGFvIII peptides per 390 kD molecule of KLH) without exogenous adjuvant. Anti-peptide IgG antibody responses were measured by end-point dilution ELISA, one, two, or three weeks after the immunization. Each dot represents an individual mouse and lines represent the geometric mean of each group.

**Figure 2:**
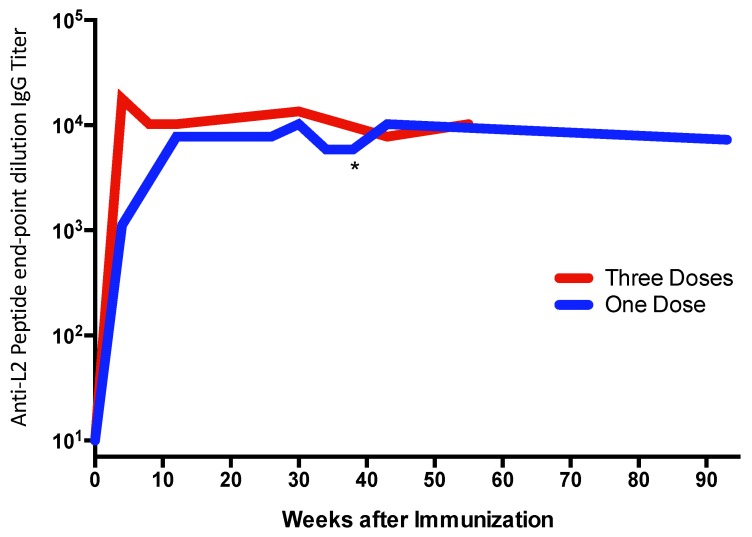
Mice were immunized with one (blue line; immunized at week zero) or three (red line; immunized at weeks zero, four, and eight) doses of 10 µg of recombinant MS2 bacteriophage VLPs displaying a peptide derived from the L2 minor capsid protein of HPV16 without exogenous adjuvant. Anti-peptide IgG antibody responses were measured by end-point dilution ELISA. The line represents the geometric mean of each group (*n* = 5/group). One of the mice in the “One Dose” group died at week 41 (indicated with an asterisk) for a reason that was unrelated to the vaccine. Antibody levels from the first six months after vaccination in the “One Dose” group were originally reported in Reference [42].

The ability of multivalent display to enhance target antigen immunogenicity is well established. Figure 1 and Figure 2 illustrate the potent immunogenicity conferred by VLP display. In Figure 1, we assessed the immunogenicity of a peptide representing a neo-epitope specific to a mutated version of the epidermal growth factor receptor (EGFRvIII) that is particularly prevalent in glioblastoma multiforme brain cancers and is associated with poor outcomes [40]. The EGFRvIII peptide was chemically conjugated either to Qß bacteriophage VLPs or to a commonly used carrier protein, keyhole limpet hemocyanin (KLH), and then used to immunize mice. After a single dose administered without exogenous adjuvant, we showed that the VLP-based immunogen elicited higher titer anti-peptide IgG antibody responses than the KLH-conjugated antigen, and that these antibody responses were elicited more rapidly (Figure 1), despite using a 5-fold lower dose of the VLP-conjugated material. Using VLPs, anti-peptide IgG was detected as early as one week after immunization. Moreover, remarkably stable levels of antibody are induced using VLP-based immunogens. In a separate study, shown in Figure 2, we longitudinally measured antibody responses in groups of mice immunized with recombinant MS2 bacteriophage VLPs, displaying a broadly neutralizing epitope from the HPV type 16 minor capsid protein (L2; the vaccine is described in [41]). Mice were given one or three doses of vaccine and then were followed for nearly two years after vaccination. Three doses elicited higher antibody titers than a single dose, but this difference became less pronounced over time. Notably, even a single dose gave remarkably stable antibody titers; levels were virtually unchanged for nearly two years after vaccination (essentially the life span of the mouse). This is likely due to the potent induction of LLPCs.

Human clinical data from trials of the VLP-based HPV vaccines Cervarix (GlaxoSmithKline) and Gardasil (Merck) also strongly support the concept that VLPs can efficiently induce LLPCs. A longitudinal study of antibody responses induced by these vaccines [43] revealed that both vaccines induce anti-HPV antibody responses that peak approximately one month after the third dose. After an initial period in which antibody levels decay rapidly (with a half-life of ~3.6 months, probably reflecting the loss of short-lived plasmablasts), these levels stabilize—there was no evidence of a further decline in antibody titers over a 4-year follow-up period. Even a single dose of HPV VLPs elicits exceedingly long-lived antibody responses. A subset of women enrolled in the phase III Costa Rica Vaccine Trial of Cervarix received just a single dose of the vaccine (this was due to various reasons, but most commonly these women discovered that they were pregnant after they received their first dose). Compared to the usual three-dose regimen, a single dose gave lower anti-HPV antibody levels, but the profile of antibody decay in the two groups was quite similar [44]. Antibody levels over the six-year period spanning one to seven years after vaccination were extraordinarily stable; these antibodies were essentially maintained at a constant level during this period. Moreover, no HPV16 or HPV18 infections were detected in this group of women during the observation period [45]. Based on these results, the US National Cancer Institute, in collaboration with a Costa Rican partner, is currently conducting a large randomized, controlled trial (the ESCUDDO study) to assess the immunogenicity and efficacy of a single-dose vaccine regimen. 

The longevity of antibody responses elicited by the HPV vaccine parallels the humoral immune responses generated by other multivalent viral vaccines. In a seminal study, Amanna and colleagues longitudinally measured human antibody responses to a diverse panel of vaccine antigens, including viral (i.e., multivalent) vaccines and non-viral (toxoid; monovalent) vaccine antigens [46]. They found that the antibodies elicited by multivalent antigens were extremely durable—for example, antibodies against measles had a calculated half-life of ~3000 years—whereas the half-life of antibodies against the monomeric tetanus and diphtheria toxoid vaccines was much shorter (~11–19 years). Interestingly, there was no relationship between the frequency of vaccine-elicited memory B cells and antibody levels, suggesting that LLPCs were elicited upon the initial exposure to the antigen, and were not due to re-activation of memory cells. Taken together, these data strongly support the idea that multivalent vaccines can elicit long-lasting responses in humans (for a review of these and other human studies, see Reference [47]).

## 4. The Special Case of Using Multivalent Vaccines to Break B-Cell Tolerance

**Figure 3:**
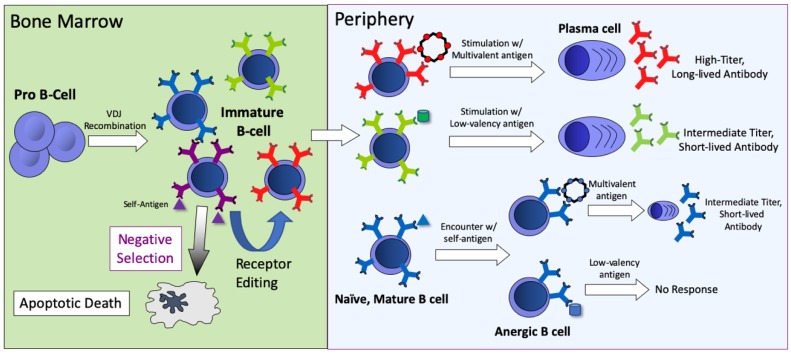
An overview of B-cell development, B-cell tolerance mechanisms, and the outcomes of stimulation with different forms of antigen (self-, low-valency, or multivalent antigen). B-cell responses are dependent on the nature of the antigen and the stage of B-cell development. In the bone marrow, immature B cells that encounter self-antigen either undergo apoptosis or receptor editing, which potentially alters receptor specificity. In the periphery, B cells that encounter multivalent antigens respond strongly, leading to the production of LLPCs that produce large amounts of antibody. Stimulation with low-valency antigens leads to lower titer and less durable antibody responses. If naïve B cells encounter self-antigen they become anergized. Anergic B cells do not respond to stimulation with low-valency antigen, but can be activated by multivalent antigens.

One of the most striking features of VLP display is its ability to elicit antibody responses against self-antigens. The immune system has erected a set of barriers that normally prevent the induction of autoantibody responses. During the early stages of B-cell development in the bone marrow, central B-cell tolerance mechanisms act to change the specificity (through a process referred to as receptor editing) or eliminate (through apoptosis) a percentage of potentially autoreactive B cells (shown schematically in Figure 3). However, these mechanisms are inefficient, and as a consequence a substantial percentage of naïve B cells that move into the periphery are potentially self-reactive [48,49]. Fortunately, when these self-reactive B cells are exposed to a soluble self-antigen in the periphery in the absence of T help, they undergo a number of changes that establish a state of unresponsiveness to subsequent antigen stimulation, known as anergy. Relative to non-anergic cells, anergic B cells are defined by decreased BCR surface expression, competitive exclusion from lymphoid follicles, and a short half-life [50,51]. Nevertheless, even these B cells are susceptible to activation by multivalent antigens. In a seminal study, Bachmann and colleagues showed that B cells from transgenic mice expressing a soluble form of the vesicular stomatitis virus glycoprotein (VSV-G) responded to vaccination with particulate, ordered forms of VSV-G (such as inactivated virions), but not to immunization with soluble monomeric VSV-G. This study suggested that multivalent antigens could effectively activate anergic B cells. Subsequent studies demonstrated that vaccines consisting of self-antigens arrayed on the surface of the VLPs could effectively induce anti-self antibody responses [52,53], that this ability is critically dependent on the density of the self-antigens displayed on VLPs [54,55], and that these interactions may be mediated by binding to surface-expressed IgD on naïve B cells [56]. These findings have led to the development of vaccine candidates that induce antibodies against self-antigens involved in chronic diseases, including angiotensin II (hypertension) [57], PCSK9 (cardiovascular disease) [58], amyloid-beta and hyper-phosphorylated tau (Alzheimer’s Disease) [59,60], and others [61]. Several of these vaccines have been tested in human clinical trials [62].

One interesting observation from human clinical trials of VLP-based vaccines targeting amyloid-beta [63] and angiotensin II [64] was that the half-life of the antibodies induced against self-antigens was fairly short, 15–20 weeks. This is in contrast to the durable antibody responses that are observed in vaccination studies using VLPs that target pathogen-derived (foreign) epitopes. Why do VLP-based vaccines that target foreign antigens strongly elicit LLPCs, but generate more transient antibody responses when targeting self-antigens? Studies comparing the reactivity of anergic and non-anergic transgenic B cells may provide an explanation. B cells respond to antigenic stimulation by upregulating a suite of molecules important in activating (i.e., CD86) and receiving help from (CD40) T helper cells. We showed that upregulation of these molecules upon stimulation by VLPs is attenuated in anergic transgenic B cells, relative to non-anergic cells [24]. It is possible that the downregulated expression of the BCR on anergic cells may account for these attenuated responses. Lower levels of BCR are likely to reduce the potential for extensive BCR crosslinking and, consequently, lead to weaker B-cell activation. Alternatively, anergic B cells may have other defects in the signaling pathways involved in B-cell activation. Regardless of the mechanism, these data indicate that reduced B-cell activation has profound downstream consequences. Weaker stimulation results in reduced numbers of LLPCs and less durable antibody responses.

## 5. Conclusions

VLPs have been exploited as a platform technology to develop vaccines for diverse targets, including specific antigens or epitopes derived from infectious disease targets, self-antigens involved in chronic diseases, and even small molecules, such as drugs of abuse [65]. In general, VLP display is regarded as a method for enhancing the magnitude of an antibody response, similar to how adjuvants can increase the immunogenicity of vaccines. However, as described in this article, and as summarized in Figure 3, the multivalent nature of VLPs also confers special immunostimulatory properties not typically conferred by adjuvants, particularly the ability to elicit durable antibody responses through the induction of LLPCs. For many targets, the induction of long-lived antibody responses is likely to be a key factor in vaccine efficacy. A deeper understanding of the mechanisms whereby multivalent antigens can lead to the induction and maintenance of LLPCs will influence vaccine design and likely lead to improved vaccines in the future.
